# APOBEC3G-UBA2 fusion as a potential strategy for stable expression of APOBEC3G and inhibition of HIV-1 replication

**DOI:** 10.1186/1742-4690-5-72

**Published:** 2008-08-04

**Authors:** Lin Li, Dong Liang, Jing-yun Li, Richard Y Zhao

**Affiliations:** 1 Department of Pathology, University of Maryland, 10 South Pine Street, MSTF700A, Baltimore, MD 21201, USA; 2 Department of Microbiology-Immunology, University of Maryland, 10 South Pine Street, MSTF700A, Baltimore, MD 21201, USA; 3 Institute of Human Virology, University of Maryland, 10 South Pine Street, MSTF700A, Baltimore, MD 21201, USA; 4 AIDS Research Department, Beijing Institute of Microbiology and Epidemiology, Beijing 100071, PR China

## Abstract

**Background:**

Although APOBEC3G protein is a potent and innate anti-HIV-1 cellular factor, HIV-1 Vif counteracts the effect of APOBEC3G by promoting its degradation through proteasome-mediated proteolysis. Thus, any means that could prevent APOBEC3G degradation could potentially enhance its anti-viral effect. The UBA2 domain has been identified as an intrinsic stabilization signal that protects protein from proteasomal degradation. In this pilot study, we tested whether APOBEC3G, when it is fused with UBA2, can resist Vif-mediated proteasomal degradation and further inhibit HIV-1 infection.

**Results:**

APOBEC3G-UBA2 fusion protein is indeed more resistant to Vif-mediated degradation than APOBEC3G. The ability of UBA2 domain to stabilize APOBEC3G was diminished when polyubiquitin was over-expressed and the APOBEC3G-UBA2 fusion protein was found to bind less polyubiquitin than APOBEC3G, suggesting that UBA2 stabilizes APOBEC3G by preventing ubiquitin chain elongation and proteasome-mediated proteolysis. Consistently, treatment of cells with a proteasome inhibitor MG132 alleviated protein degradation of APOBEC3G and APOBEC3G-UBA2 fusion proteins. Analysis of the effect of APOBEC3G-UBA2 fusion protein on viral infectivity indicated that infection of virus packaged from HEK293 cells expressing APOBEC3G-UBA2 fusion protein is significantly lower than those packaged from HEK293 cells over-producing APOBEC3G or APOBEC3G-UBA2 mutant fusion proteins.

**Conclusion:**

Fusion of UBA2 to APOBEC3G can make it more difficult to be degraded by proteasome. Thus, UBA2 could potentially be used to antagonize Vif-mediated APOBEC3G degradation by preventing polyubiquitination. The stabilized APOBEC3G-UBA2 fusion protein gives stronger inhibitory effect on viral infectivity than APOBEC3G without UBA2.

## Background

There is an active and antagonistic host-pathogen interaction during HIV-1 infection. Upon infection by HIV-1, host cells react with various innate, cellular and humoral immune responses to counteract the viral invasion. Limited and transient restriction of viral infection is normally achieved. However, HIV-1 overcomes these antiviral responses through various counteracting actions. For example, APOBEC3G (apolipoprotein B mRNA-editing enzyme catalytic polypeptide-like 3G), a host innate antiviral protein [1], was found to be responsible for the inhibition of Vif-minus-HIV-1 infection [2]; whereas Vif counteracts this host cellular response by promoting proteasome-mediated degradation of APOBEC3G [3].

APOBEC3G is a member of cellular cytidine deaminase family. At the late phase of viral life cycle, APOBEC3G is encapsided into the virus particles through interaction with viral Gag protein [4-8]. Specifically, N-terminal domain of APOBEC3G is known to be important for targeting the protein to viral nucleoprotein complex and confers antiviral activity [9]. Once a virus enters a new cell, virus genomic RNA will be reverse transcribed into cDNA before integrating into the host cellular chromosome DNA. As part of the host innate immune responses, APOBEC3G prevents viral cDNA synthesis by deaminating deoxycytidines (dC) in the minus-strand retroviral cDNA replication intermediate [10-14]. As result, it creates stop codons or G-A transitions in the newly synthesized viral cDNA that is subjective to elimination by host DNA repair machinery [12,14]. As part of the viral counteracting effort, HIV-1 Vif counteracts this innate host cellular defense by promoting its degradation through proteasome-mediated proteolysis [3,15-18]. Specifically, Vif recruits Cullin5-EloB/C E3 ligase to induce polyubiquitination of APOBEC3G [19,20]. Specifically, Vif uses a viral SOCX-box to recruit EloB/C [12] and a HCCH motif to recruit Cullin 5 [21]. By eliminating APOBEC3G from the cytoplasm, Vif prevents APOBEC3G from packaging into the viral particles thus augment HIV-1 infection in "non-permissive" cells [2]. Based on the Vif-APOBEC3G antagonism at the protein level, it is conceivable that creation of proteolysis-resistant APOBEC3G could potentially strengthen the host innate anti-viral response and further inhibit HIV-1 infection. The objective of this pilot study was to test this premise.

Ubiquitin-associated domain 2 (UBA2) is typically 45 amino acids long that specifically bind to both mono- and polyubiquitins [22]. Homonuclear NMR spectroscopy revealed that UBA2 domain contains a low resolution structure composed of three α-helices folded around a hydrophobic core [23], suggesting that UBA2 domain may be involved in multiple functions. Indeed, functions of UBA2 have been linked to protein ubiquitination, UV excision repair, and cell signaling [24]. For example, UBA2 domain is found in a family of protein including human HHR23A, budding yeast Rad23 and fission yeast Rhp23 [22,25]. All of the HHR23A homologues are composed of an N-terminal ubiquitin-like (UBL) domain and two ubiquitin-associated (UBA) domains, i.e., an internal UBA1 domain and a C-terminal UBA2 domain [22]. HHR23A interacts with 26S proteasome through its N-terminal UBL domain to promote protein degradation [26-28]. UBA domains bind to ubiquitin [29-31] and play a role in targeting ubiquitinated substrates to the proteasome [32-34]. As a general rule, ubiquitination of proteins and subsequent recruitment of ubiquitinated proteins to the proteasome always results in rapid degradation of those proteins [35]. However, binding of HHR23A or Rad23 to ubiquitin and proteasome does not lead to their degradation [26,36]. It was believed that there must be a specific domain in the HHR23A or its homologous proteins that serve as a protective "stabilization signal" and prevents them from proteasome-mediated proteolysis [37]. Indeed, UBA2 domain was recently found to function as a cis-acting and transferable "stabilization signal" [35]. This "stabilization signal" can be destroyed simply by introducing a point mutation at residue 392 (L392A) of the UBA2 domain [35].

Since Vif promotes APOBEC3G degradation through proteasome-mediated proteolysis of ubiquitinated proteins, and because UBA2 decreases protein degradation through this pathway, we hypothesize that UBA2, if fused with APOBEC3G, should be able to act as a "stabilization signal" and to protect APOBEC3G from Vif-mediated degradation. Here we tested this hypothesis by comparing protein stability of normal APOBEC3G protein with the APOBEC3G-UBA2 fusion proteins in the presence of Vif. To gain additional functional insights into the molecular mechanism underlying the ability of UBA2 to prevent protein degradation, the effects of UBA2 on APOBEC3G protein degradation under the conditions of excessive polyubiquitination or the lack of proteasome activity were examined. The effect of UBA2 on APOBEC3G stability and its impact on viral infectivity was also investigated.

## Results

### APOBEC3G fused with UBA2 is more resistant to Vif-mediated protein degradation than APOBEC3G

**Figure 1:**
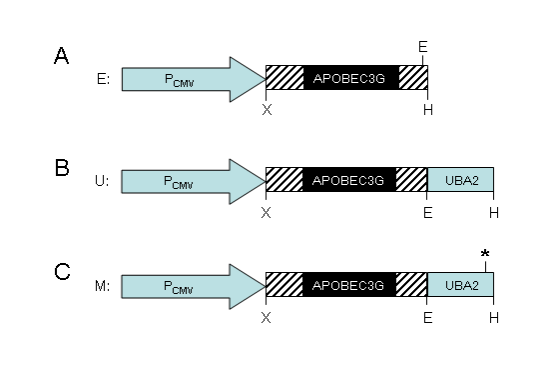
**Schematic drawings of the APOBEC3G-carrying plasmids**. E: untagged APOBEC3G-carrying plasmid (pcDNA3.1(-)-Apo-E/Hygromycin); U: same plasmid but contains an in-frame fusion of UB2A with APOBEC3G (pcDNA3.1(-)-Apo-U/Hygromycin); M: same as U but contains an in-frame fusion of a mutated UBA2* with APOBEC3G (pcDNA3.1(-)-Apo-M/Hygromycin). The asterisk * by UBA2 indicates location of a single point mutation in the UBA2 domain (L392A) that renders it incapable of stabilizing proteins [35]. P_CMV_, CMV promoter; the single letter restriction enzyme designations are: X, *XhoI*; E, *EcoRI*; H, *HindIII*.

**Figure 2:**
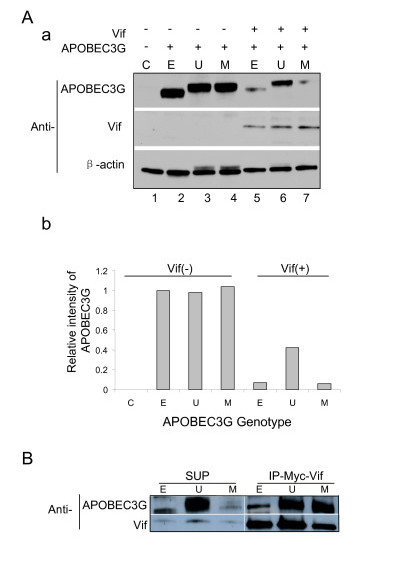
**APOBEC3G fused with UBA2 domain is more resistant to Vif-mediated degradation than APOBEC3G**. A-a. HEK293 cells, which is APOBEC3G-negative, was co-transfected with 1.5 μg of Vif-carrying plasmid (Vif-VR1012) DNA and 6 μg of plasmid DNA that expresses untagged APOBEC3G (E), APOBEC3G-UBA2 (U) fusion protein or APOBEC3G-UBA2* mutant fusion protein (M), respectively. Forty-five hours post-transfection (p.t.), cell lysates were subject to SDS polyacryladmide gel electrophoresis and analyzed by Western blot analysis using monoclonal anti-APOBEC3G and anti-Vif antibodies. Level of protein loading was measured by anti-β-actin antibody. A-b. The intensity of APOBEC3G protein was determined by densitometry. Value of the relative intensity of APOBEC3G was calculated in relative to the untagged APOBEC3G (E) and adjusted based on the relative intensity of β-actin in each lane to that of the control (C). B. UBA2 fusion to APOBEC3G does not affect its binding to Vif. Myc-tagged Vif was pulled-down in different APOBEC3G-producing HEK293 cells by immunoprecipitation using anti-Myc antibody. Binding of different forms of APOBEC3G to Vif was detected by using anti-APOBEC3G and anti-Vif antibodies, respectively. SUP, supernatants; IP, immunoprecipitation.

To test whether UBA2 can stabilize APOBEC3G protein, UBA2 was fused at the C-terminal end of APOBEC3G (Fig. 1A). The APOBEC3G without the UBA2 fusion (Fig. 1B) or fused with a mutant L392A UBA2 that is incapable of stabilizing proteins (Fig. 1C; [35]), was used as controls. The fusion products were cloned into a mammalian gene expression plasmid pCDNA3.1 and the resulting plasmids were designated as pcDNA3.1(-)-Apo-E/Hygromycin (E) for the untagged APOBEC3G, pcDNA3.1(-)-Apo-U/Hygromycin (U) for the APOBEC3G-UBA2 fusion, and pcDNA3.1(-)-Apo-M/Hygromycin (M) for the APOBEC3G-UBA2* mutant fusion. Protein stability of APOBEC3G was determined either by expression of these plasmids individually or by co-transfection of each individual APOBEC3G-carrying plasmid construct with a Vif-carrying plasmid (Vif-VR1012) in HEK293 cells. As shown in Fig. 2A, expression of untagged APOBEC3G produced a strong protein band at approx. 46 kD consistent with the size of APOBEC3G (Fig. 2A, lane 2). Slight increase in molecular weight was detected in the APOBEC3G-UBA2 and APOBEC3G-UBA2* fusion products (Fig. 2A, lanes 3–4).

Approximately equal amount of protein was produced in each of these plasmid constructs without *vif *gene expression (Fig. 2A–b). When *vif *is expressed in the APOBEC3G-producing HEK293 cells, a significant decrease of APOBEC3G with more than 10-fold reduction was noticed in the untagged APOBEC3G cells (Fig. 2A, lane 5). In contrast, a small with about 2-fold decease of APOBEC3G-UBA was detected when APOBEC3G was fused with the wild type UBA2 (Fig. 2A, lane 6). Consistent with the finding that a single point mutation of APOBEC3G (L392A) abolishes the ability of APOBEC3G to stabilize proteins [35], production of Vif in these cells reduced the APOBEC3G-UBA2* protein level to the level that is similar to the untagged APOBEC3G (Fig. 2A lane 7 *vs*. lane 5). Together, these data suggested that the wild type UBA2, when it is fused with APOBEC3G, is indeed able to stabilize APOBEC3G and renders it more resistant to Vif than the untagged APOBEC3G.

One possibility for the observed resistance of APOBEC3G-UBA2 to Vif could be explained by the reduced binding of APOBEC3G-UBA2 to Vif. To test this possibility, Myc-tagged Vif was pull-down by immunoprecipitation in the APOBEC3G-producing HEK293 cells. Western blot analyses were carried out to measure the bindings of different APOBEC3G constructs to Vif. As shown in Fig. 2B, no obvious reduction of the binding of APOBEC3G-UBA2 to Vif was observed (Fig. 2B, lane 5). In fact, binding of APOBEC3G-UBA2 to Vif appeared to be stronger than the untagged APOBEC3G or APOBEC3G with the mutated UBA2. This increase binding could potentially be due to presence of the excessive APOBEC3G-UBA2, which is clearly shown by the high level of APOBEC3G remained in the supernatant (Fig. 2B, lane 2). Nevertheless, these data suggest that the observed resistance of APOBEC3G to Vif is not caused by reduction binding.

### Overexpression of polyubiquitin diminishes the ability of UBA2 to stabilize APOBEC3G against Vif

**Figure 3:**
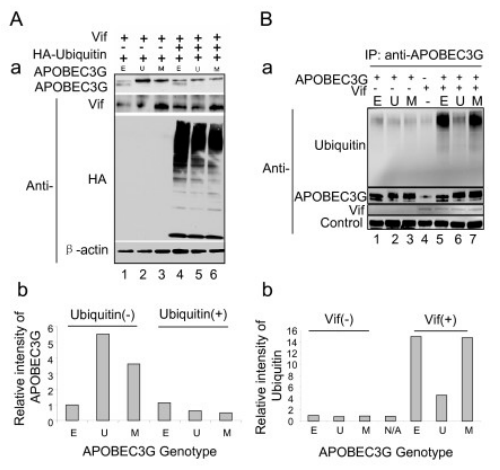
**Fusion of UBA2 to APOBEC3G limits its polyubiquitination**. A-a, Expression of polyubiquitin abolishes the ability of UBA2 to stabilize APOBEC3G. HEK293 cells were co-transfected as described in Fig. 2. In addition, 3 μg of a plasmid DNA (pcDNA3.1-HA-Ubiquitin) that produces polyubiquitin [40,41] was also co-transfected to HEK293 cells. Western blot analysis was carried out by using monoclonal anti-APOBEC3G, anti-Vif, anti-HA, and anti-β-actin antibodies respectively. A-b. The intensity of APOBEC3G protein and value of the relative intensity of APOBEC3G was determined as described in Fig. 2. Note, a protein band that migrates with similar size to APOBEC3G-UBA2 as shown in lane E sometimes react to anti- APOBEC3G antibody. This is a non-specific protein band because it only reacts to certain batches of anti-APOBEC3G antibody. To eliminate this background, the protein intensity of APOBEC3G-UBA2 and APOBEC3G-UBA2* was calculated by subtracting the level of this non-specific protein. B. Fusion of UBA2 to APOBEC3G shows reduced polyubiquitination. B-a, Vif protein was pull-down in different APOBEC3G-producing HEK293 cells by immunoprecipitation using anti-Vif antibody. Binding of high molecular weight of ubiquitin (polyubiquitin) to Vif was detected by using anti-ubiquitin antibody. B-b, the relative intensity of ubiquitin to β-actin control was determined by densitometry. Also note that there are not much protein level differences of APOBEC3G between lane 2 (U) and lane 3 (M). This is likely due to the fact that more protein is loaded in lane 3 than lane 2 as shown by the relative protein levels of β-actin.

Most cellular proteins are targeted for degradation by the proteasome. Prior to proteasome-mediated proteolysis, the proteins are covalently attached to ubiquitin. A poly-ubiquitin chain will be formed and function as a degradation signal. The poly-ubiquitinated protein can then be recognized by the 26S proteasome for degradation [38]. If the ubiquitin chain elongation is interrupted, this protein cannot be recognized by the 26 S proteasome and thus it cannot be degraded. UBA2 binds to ubiquitin directly and inhibits elongation of polyubiquitin chains by capping conjugated ubiquitin [30,39]. Since Vif mediates APOBEC3G degradation by promoting protein ubiquitination of APOBEC3G [3]*via *Cullin5-EloB/C E3 ligase to induce polyubiquitination of APOBEC3G [19,20], it is possible that UBA2 may either sequester ubiquitin from APOBEC3G or prevent polyubiquitin chain elongation. As results, the un-ubiquitinated APOBEC3G becomes resistant to proteasome-mediated proteolysis. To test this possibility, polyubiquitin was overproduced through a pcDNA3.1-HA-Ubiquitin plasmid [40,41] in the HEK293 cells co-producing Vif and various APOBEC3G products. As shown in Fig. 3A, APOBEC3G-UBA2 fusion protein showed relative strong intensity in comparison with the untagged APOBEC3G (Fig. 3A–a, lane 3 *vs*. lane 1). However, production of excessive polyubiquitin completely abolished the difference between the protein level of APOBEC3G-UBA2 and APOBEC3G (Fig. 3A–a, lane 5 *vs*. lane 4). Western protein blotting with anti-Vif and anti-HA for ubiquitin detection confirmed proper production of Vif and polyubiquitin in these cells. Therefore, over-production of polyubiquitin can diminish the ability of UBA2 for APOBEC3G stabilization.

To further verify whether fusion of APOBEC3G to UBA2 results in less binding to polyubiquitin, APOBEC3G in the presence or absence of Vif was collected by immunoprecipitation using anti-APOBEC3G monoclonal antibody. The pull-down protein products were subject to Western blot analyses as shown in Fig. 3B. Approximately equal amount of APOBEC3G was collected in all cells with the exception of the control cells (Fig. 3B–a, lane 4), in which only endogenous APOBEC3G was pull-down. Without Vif, minimal and background level of polyubiquitin was detected in all APOBEC3G-producing cells (Fig. 3B–a, lanes 1–3). In contrast, strong polyubiquitin was detected in the *vif*-expressing cells with untagged APOBEC3G or APOBEC3G-UBA2* (Fig. 3B–a, lanes 5 and 7). However, much reduced level of polyubiquitination was observed in *vif*-expressing cells carrying the APOBEC3G-UBA2 (Fig. 3B–a, lane 6). This observation provides direct support to the notion that UBA2 may prevent polyubiquitin chain elongation on APOBEC3G.

### Treatment of HEK293 cells with proteasome inhibitor MG132 alleviated degradation of APOBEC3G and APOBEC3G-UBA2 fusion proteins

**Figure 4:**
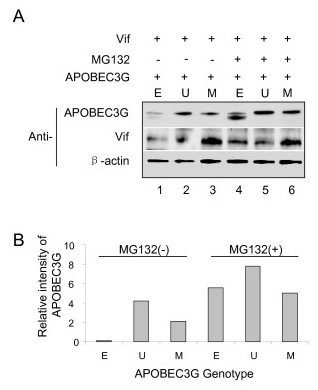
**Treatment of HEK293 cells with proteasome inhibitor MG132 alleviates degradation of APOBEC3G and APOBEC3G-UBA2 fusion proteins**. HEK293 cells were co-transfected as described in Fig. 2. Transfected cells were treated with 2.5 mM of the proteasome inhibitor MG132 27 hrs *p.t*. Western blot analysis was carried out as described in Fig. 3. B. The intensity of APOBEC3G protein and the value of the relative intensity of APOBEC3G were calculated the same way as described in Fig. 2.

To test whether inhibition of the 26S proteasome activity has any impact on the ability of UBA2 to stabilize APOBEC3G against Vif, APOBEC3G-producing HEK293 cells were treated the proteasome inhibitor MG132 in the presence of Vif. APOBEC3G protein levels were measured and compared between cells with or without the MG132 treatment. Similar to what we have shown in Fig. 2A, the protein intensity of APOBEC3G-UBA2 was significantly higher than that without the UBA2 tag (Fig. 4A, lane 2 *vs*. lane 1), suggesting the protein stabilizing capacity of UBA2. APOBEC3G fusion with a mutant UBA2* reduced its ability to stabilize APOBEC3G (Fig. 4A, lane 3). Significantly, HEK293 cells treated with the proteasome inhibitor MG132 all showed much higher protein intensities than the APOBEC3G-UBA2 producing cells without MG132 treatment (Fig. 4A, lanes 4–6 *vs*. lane 2). These enhanced protein levels were observed in all of the APOBEC3G protein constructs regardless whether it is fused with UBA2 or not, suggesting UBA2 stabilizes APOBEC3G through resistance to proteasome-mediated proteolysis.

### Viruses packaged from cells expressing APOBEC3G-UBA2 fusion protein gives stronger suppressive effect on viral infectivity than that packed from APOBEC3G

**Figure 5:**
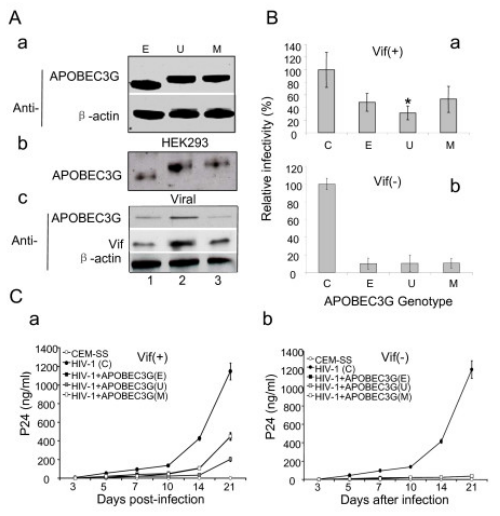
**APOBEC3G fused with UBA2 confers stronger suppressive effect on viral infectivity than APOBEC3G**. A. Packaging of different APOBEC3G variants into HIV-1 viral particles from HEK293 cells that stably express high level of APOBEC3G, APOBEC3G-UBA2 or APOBEC3G-UBA2*. a, The HEK293 cells that stably producing high level of APOBEC3G, APOBEC3G-UBA2 or APOBEC3G-UBA2* were established by selection of hygromycin resistant cells (300 μg/ml) for 2 weeks and verified by the Western blot analyses; b, Different APOBEC3G variants were equally packaged into the HIV-1 viral particles and harvested from HEK293 cells by expressing pNL4-3 plasmid in the control HEK293 cells lack of APOBEC3G (C) or HEK293 cells stably expressing different APOBEC3G variants; c, effect of viral expressing Vif on protein degradation of APOBEC3G variants. B. Effect of APOBEC3G variants on viral infectivity in MAGI-CCR5 cells. The MAGI-CCR5 cells were infected with viral supernatants harvested from the HEK293 cells that stably produce either no APOBEC3G or high level of different APOBEC3G constructs. Forty-eight hours post-infection, cells were stained by β-galactosidase for HIV-infected cells as described previously [43]. The viral infectivity of APOBEC3G-negative control HEK293 cells (C) was calibrated to 100% for comparison purpose. The viral infectivity was determined by comparing the total number of blue cells with the total number of cells counted. Data shown represent average of three independent experiments. Error Bars shown are standard errors of the means. a. results of wild type Vif(+) HIV-1_NL4-3 _infection; b. results of Vif(-) HIV-1_NL4-3ΔVif _infection. * *p *< 0.01. C. Effect of APOBEC3G variants on spread viral infection in CEM-SS cells. CEM-SS cells expressing APOBEC3G(E), APOBEC3G-UBA2(U), or APOBEC3G-UBA2(M) was infected by Vif(+) or Vif(-) HIV-1_NL4-3_. P24 antigen was measured post-infected day 3, 5, 7, 10 14, 21. a. results of wild type Vif(+) HIV-1_NL4-3 _infection; b. results of Vif(-) HIV-1_NL4-3 Δ Vif _infection.

To test whether APOBEC3G stabilized by UBA2 can further enhance the suppressive effect of APOBEC3G on viral infectivity, the HIV-1 viral particles were produced from HEK293 cells that expressing different constructs of APOBEC3G as described. To minimize potential differences of production of each protein construct and viral packaging, HEK293 cells that stably express APOBEC3G, APOBEC3G-UBA2, and APOBEC3G-UBA2* fusion proteins were created by proper antibiotic selection. High level expression of these proteins was further verified by Western blot analysis (Figure 5A–a). To produce APOBEC3G-carrying viral particles, the pNL4-3 plasmid was expressed in HEK293 viral producing cells that stably expressing different APOBEC3G fusion proteins. The infectious viral particles were harvested 48 hrs after transfection as previously described [42]. Presence of different APOBEC3G constructs was detected with approx. equal amount within all three types of viral particles (Fig. 5A–b). To test whether the potential effect of the viral expressing Vif on the stability of APOBEC3G, levels of APOBEC3G in the viral particle producing HEK293 cells were further measured after viral gene expressions. Essentially the same Vif effect on APOBEC3G was seen between the viral expressing Vif and Vif expressed from a plasmid (Fig. 5A–c).

To test the potential impact of different APOBEC3G constructs packaged in the viral particles on viral infectivity and replication, concentration of the viral stocks were normalized by determination of the p24 antigen levels. The viral infectivity of viruses packaged with different APOBEC3G constructs were measured with the MAGI-CCR5 assay as previously described [43]. This assay measures viral infectivity in a single cycle of viral infection. About 50% reduction of viral infectivity was observed in viruses packed from cells producing high level of APOBEC3G than endogenous level of APOBEC3G (Fig. 5B–a, lane E *vs*. lane C). An additional 17% and significant reduction of viral infectivity (P < 0.01) was also observed in viruses packaged from cells expressing the APOBEC3G-UBA2 fusion protein (Fig. 5B–a, lane U). In contrast, no significant difference was detected between viruses carrying untagged APOBEC3G or APOBEC3G fused with a mutant UBA2, indicating the additional reduction of viral infectivity observed in the APOBEC3G-UBA2 fusion was indeed due to the stabilizing effect of UBA2 on APOBEC3G (Fig. 5B–a, lane M *vs*. lane E). These differences in viral infectivity were not observed in the Vif(-) viral infections suggesting the observed differences were caused by Vif (Fig. 5B–b).

To further evaluate the observed effects of APOBEC3G variants on spread viral infection, CEM-SS cells, a cell line derived from CD4-positive T-lymphocytes, were infected with the same Vif(+) and Vif(-) viral particles packaged with different APOBEC3G variants as described above. P24 antigenemia was measured from day 3 to day 21 post-viral infection. Similar suppressive effects of the APOBEC3G variant on viral infection as described above for the MAGI-CCR5 experiment were also seen in CEM-SS cells (Fig. 5C). The differences are however most pronounced in day 21 post-infection: while infection of CEM-SS with the control viral particles produced approximately 1,200 ng/ml of p24 antigen, about 400 ng/ml of p24 antigen was seen in CEM-SS cells infected with viral particles packed with either untagged APOBEC3G or the UBA2 mutant variant (Fig. 5C–a). Additional reduction of viral replication with approx. 200 ng/ml was observed in the same cells when they were infected with the viral particles packaged with the APOBEC3G-UBA2. All of the APOBEC3G variants conferred the same level of strong viral suppression against Vif(-) viral infection (Fig. 5C–b), suggesting that the observed differences as described in the Vif(+) viral infections were due to interaction between Vif and APOBEC3G.

## Discussion

In this report we demonstrated, proof of principle, a plausible strategy that could be used to stabilize APOBEC3G and to further reduce viral infection. Consistent with a previous study [44], we first confirmed that virus packaged from the HEK293 cells expressing high level of APOBEC3G gives stronger suppressive effect on viral infectivity than the virus that was packaged from normal cells (Fig. 5B, lane C *vs*. E). Moreover, we showed that APOBEC3G protein, when it is fused with an ubiquitin-associated domain, i.e., UBA2, becomes more resistant to Vif-mediated protein degradation (Fig. 2A). Importantly, additional suppression of viral infectivity or replication was found in the APOBEC3G-UBA2-carrying virus in comparison with the APOBEC3G-carrying virus without the UBA2 fusion (Fig. 5B–C). The observed suppression of APOBEC3G-UBA2 on viral infection was diminished in Vif(-) viral infections suggesting that the observed APOBEC3G-UBA2 effect was due to its interaction with Vif. Interestingly, despite its resistance of APOBEC3G-UBA2 to Vif-induced degradation, APOBEC3G-UBA2 is packaged at essentially the same level into wild type HIV-1 virions as untagged APOBEC3G or APOBEC3G tagged with mutant UBA2 (Fig. 5). This observation seems to argue against the dogma that Vif prevents packaging of APOBEC3G by inducing its proteasomal degradation. Moreover, the wild type HIV-1 produced in the presence of APOBEC3G-UBA2 appeared to be more infectious than the Vif(-) mutant (Fig. 5B a–b [U]). This finding could potentially be even more significant than the reduction in infectivity of the wild type virus "E" *vs*. "U" as shown in Fig. 5Ba, as it may indicate that Vif may confer suppressive effect on APOBEC3G. in addition to the degradation effect. Indeed, a recent report by Opi et al. [45] showed that inhibition of viral infectivity by a degradation-resistant form of APOBEC3G is still sensitive to Vif. Together these data suggest that stabilized APOBEC3G by UBA2 may have contributed to the observed viral suppression. This premise is certainly supported by our observation that the same virus that carries APOBEC3G fused with a mutant UBA2 lost its suppressive effect on viral infectivity (Fig. 5B–C).

It should be mentioned that the observed suppressive effect of APOBEC3G-UBA2 on viral infection is not as pronounced as the suppressive effect observed in an APOBEC3G D128K mutant, in which the D128K mutant inhibits HIV-1 by several hundred fold [46,47]. One possible explanation of the discrepancy between our study and that of the cited APOBEC3G D128K study might be due to the difference in binding of Vif to these APOPEC3G variants. For example, Vif still bind to APOBEC3G-UBA2 (Fig. 2B). In contrast, Vif no longer bind to the D128K mutant [46,47].

The molecular mechanism underlying the ability of UBA2 to stabilize APOBEC3G needs to be further delineated. There are three possibilities that could potentially explain the observed stabilizing effect of UBA2 on APOBEC3G based on the published reports and data presented here. First, similar to the finding described in the budding yeast homologue (Rad23) of HHR23A [48], UBA2 prevents Rad23 protein degradation by binding to the UBL domain at its N-terminal end where the 26S proteasome attaches [35,49]. Following the same scenario, binding of UBA2 to the 26S proteasome-binding site could conceivably protect APOBEC3G from proteasome-mediated degradation. However, this possibility is unlikely because there is no UBL domain or alike which thus thus far been identified in APOBEC3G. Second, C-terminal fusion of UBA2 to APOBEC3G may stabilize APOBEC3G by hindering it from unfolding by the 19S regulatory subunit of the proteasome, a scenario that has been described previously [35]. Prior to proteasome-mediated degradation of a protein, 19S regulatory subunit of proteasome must first unfold the polyubiquitinated protein as subsequent degradation requires an unstructured initiation site of the unfolded protein [50]. An early *in vitro *study showed that tightly folded C-terminal domains can block protein unfolding and thus delay proteasomal degradation [51]. It is possible that fusion of UBA2 with APOBEC3G created a tightly folded C-terminal end of protein that block APOBEC3G-UBA2 unfolding and proteasomal degradation. If this is the case, addition of excessive ubiquitin or inhibition of proteasome activity should not affect the level of protein observed. Therefore, this possibility should be excluded. Third, UBA2 prevents polyubiquitination of APOBEC3G the same way as described for other proteins [48,52]. UBA2 inhibits elongation of polyubiquitin chains by capping conjugated ubiquitin [30,39]. Prior to proteasome-mediated proteolysis, the protein destined to be degraded is first polyubiquitined. If the ubiquitin chain elongation is somehow restricted, this protein cannot be recognized by the 26 S proteasome and thus it cannot be degraded. To a certain extent, our results seem to support this possibility because when excessive polyubiquitin were produced, it abolishes the ability of UBA2 to stabilize APOBEC3G (Fig. 3A). Furthermore, our data showed APOBEC3G-UBA2 bound less polyubiquitin than the other APOBEC3G variants (Fig. 3B). Nevertheless, should UBA2 indeed stabilized APOBEC3G through this mechanism, the stabilization to proteasome-mediated proteolysis by UBA2 is not complete because the 26S proteasome is still able to degrade part of the APOBEC3G-UBA2 protein. This was certainly supported by the observation that inhibition of the 26S proteasome activity by MG132 resulted in further increase of the APOBEC3G-UBA2 level (Fig. 4A, lane U). In order to further explore the potential ability of UBA2 to stabilize APOBEC3G, future experiments could include testing of different UBA2 constructs isolated from various species such as budding or fission yeast. Alternatively, multiple and tandem UBA2 could potentially be used to test whether they can provide stronger stabilizing effect on APOBEC3G than a single UBA2. Additionally, it should be pointed out that introduction of therapeutic APOBEC3G-UBA2 into human cells, through whatever technique, will not eliminate preexisting endogenous (untagged) APOBEC3G. Such APOBEC3G could tie up Vif and minimize degradation-independent activities of Vif thus making APOBEC3G-UBA2 more effective. This possibility can certainly be tested by co-expression of tagged and untagged APOBEC3G.

## Conclusion

This is a proof of concept study that provides, for the first time, evidence showing APOBEC3G, when it is stabilized by UBA2, attenuates HIV-1 infectivity. Further refinement of this strategy is needed to develop a more efficient way to stabilize APOBEC3G. It nevertheless promises a new and testable approach in that it may contribute to future strategies against HIV infection.

## Methods

### Cell lines and Plasmids

HEK293 cell was maintained in Dulbecco's minimal essential medium (DMEM) supplemented with 10% fetal bovine serum. MAGI-CCR5 cell, a HeLa-CD4 cell derivative that expresses CCR5 and that has an integrated copy of the HIV-1 long terminal repeat (LTR)-driven β-D-galactosidase reporter gene [43], was maintained in Dulbecco's modified Eagle's medium (DMEM) supplied with 10% (vol/vol) fetal bovine serum (FBS) (Bio Whittaker), 200 μg/ml G418, 50 U/ml hygromycin (CalBiochem), and 1 μg/ml puromycin. CEM-SS cells were grown in RPMI 1640 medium. To produce APOBEC3G, APOBEC3G-UBA2, and APOBEC3G-mutant UBA2 fusion proteins, three plasmids including pcDNA3.1(-)-Apo-E/Hygromycin (E), pcDNA3.1(-)-Apo-U/Hygromycin (U), and pcDNA3.1(-)-Apo-M/Hygromycin (M) were constructed according to the strategy shown in Figure 1. To make these plasmid constructs, the *APOBEC3G *gene was amplified from the plasmid pcDNA3.1-*HA*-*APOBEC3G *by PCR. The 5' primer used for the construction of all three plasmids was 5'-GCGCGCGCGCCTCGAGACCATGAAGCCTCACTT-3'; The 3' primers used for the construction of the E plasmid was 5'-ATCCAAGACGGAATTCCTAGAACTCGTTTTCCTGATTCTGGAG-3' and the 3' primer used for the U and M plasmid was 5'-ATCCAAGACGGAATTCGTTTTCCTGATTCTGGAG-3'. The *UBA2 *gene fragment was amplified from plasmid pcDNA3.1-*HHR23A *by PCR. The 5' primer used was 5'-ATCCAAGACGGAATTCACGCCGCAGGAGAAAGAAGCTATAG-3'; the 3' primer for the APOPEC3G-UBA2 fusion was 5'-ATCGTACTCGAAGCTTCTAACTCAGGAGGAAGTTGGCAG-3'; and the 3' primer for the APOPEC3G-UBA2* fusion was 5'-ATCGTACTCGAAGCTTCTAACTCAGagcGAAGTTGGCAG-3'. Purified PCR products were first cloned into the mammalian expression plasmid pcDNA3.1(-)/neo, the gene fragments were then cut off and cloned into a pcDNA3.1(-)/Hygromycin plasmid. Correct insertion and nucleotide sequence of each gene fragment was verified by restriction enzyme digestions and was confirmed by nucleotide sequencing. The pcDNA3.1-*HA*-*Ubiquitin *plasmid was used to express polyubiquitin [40,41]. The pNL4-3 plasmid was used to packaged virus in HEK293 cells as described previously [42].

### Immunoprecipitation and immunoblot analysis

Transfected HEK293 cells were harvested, washed 2 times with cold PBS, and lysed in lysis buffer (50 mM Tris, pH 7.5, with 150 mM NaCl, 1% Triton X-100, and complete protease inhibitor cocktail tablets) at 4°C for 1 h, then centrifuged at 10,000 g for 30 min. Cell lysates were mixed with anti-APOBEC3G Ab (NIH reagents program) and incubated at 4°C for overnight. The mixture of antigen and antibody was incubated with protein A agarose beads (Sigma) and incubated at 4°C for 3 h. Samples were then washed three times with washing buffer (20 mM Tris, pH 7.5, with 100 mM NaCl, 0.1 mM EDTA, and 0.05% Tween-20). Beads were eluted with loading buffer. The eluted materials were then analyzed by SDS-PAGE.

For Western blot analysis, HEK293 cells were harvested and rinsed with ice-cold HEPES-buffered saline (pH 7.0), then lysed in an ice-cold cell lysis buffer [20 mM Tris-HCl, pH7.6, 150 mM NaCl, 1 mM EDTA, 0.5% Nonidet P-40, 1 mM DTT, 5 μM Trichostatin A, 1 mM sodium orthovanadate, 1 mM PMSF, 1 mM NaF and complete protease inhibitors (Roche Applied Science)]. Cellular lysates were prepared and the protein concentration was determined using the Pierce protein assay kit. For immunoblotting, an aliquot of total lysate (50 μg of proteins) in 2 × SDS-PAGE sample buffer (1:1 v/v) was electrophoresed and transferred to a nitrocellulose filter. Filters were incubated with appropriate primary antibody in Tris-buffered saline (TBS, pH 7.5) and 5% skim milk or 5% BSA overnight. The primary antibodies include anti-APOBEC3G antibody at a 1:500 dilution (NIH reagents program), anti-Vif antibody at a 1:200 dilution (NIH reagents program), anti-HA antibody at a 1:1000 dilution, and anti-β-actin (Sigma) antibody at a 1:3,000 dilution. After washing, the filter was further incubated with secondary antibody in TBS-Tween-20 (TBS-T) buffer for 1 h. Protein bands were visualized by an ECL detection system. Goat anti-mouse or anti-mouse IgG-HRP conjugate (dilution of 1:3,000) were used as secondary antibodies according to the corresponding primary antibodies.

### Transfection and Viral Packaging

Plasmid DNA was transfected into HEK293 or CEM-SS cells by using Lipofectamine 2000 transfection reagent (Invitrogen) according to the manufacturer's instructions. To create stable APOBEC3G-expressing cell lines, the plasmid DNA of pcDNA3.1(-)-Apo-E/Hygromycin, pcDNA3.1(-)-Apo-U/Hygromycin, pcDNA3.1(-)-Apo-M/Hygromycin was transfected into HEK293 cells. HEK293 cells that stably produce a high level of APOBEC3G, APOBEC3G-UBA2 or APOBEC3G-UBA2* were first established by selection of hygromycin resistant cells (300 μg/ml) for 2 weeks and verified by the Western blot analyses (Fig. 5A–a). To generate infectious viral particles, HEK293 was inoculated into 6-well plate one day before pNL4-3 plasmid transfection to ensure adequate (50–60%) cell confluency. The full-length molecular clone pNL4-3 and pNL4-3 ΔVif plasmid was then transfected into HEK293 cells to produce wild type Vif(+) or mutant Vif(-) HIV-1 viruses [42]. Forty-eight hours *p.t*., HIV-1 viral particles were harvested from the supernatants of HEK293 cells by centrifugation at 1,000 rpm/min for 5 min. The isolated viral particles were split into 2 ml aliquots and stored in -80°C. To ensure equal levels of viral infection, the viral stocks were normalized by determining levels of p24 antigens in each viral stock. The level of p24 antigen was determined by using a commercial p24 antigen kit from ZeptoMetrix Co. (Buffalo, NY) following the manufacture's instructions.

### Viral infections

To evaluate the suppressive effect of APOBEC3G, APOBEC3G-UBA2 and APOBEC3G-UBA2* on viral replication in proliferating CD4+ T-lymphocytes, CEM-SS cells (APOBEC3G negative CD4+ T-lymphocytes) that stably express a plasmid control, APOBEC3G, APOBEC3G-UBA2 and APOBEC3G-UBA2* were established. 1 × 10^6 ^CEM-SS cells were either mock infected or infected with 3000 TCID50 of HIV-1_NL4-3 _and HIV-1_NL4-3ΔVif_. The viral replication was measured by p24 antigenemia.

### MAGI assay

MAGI assay was used to determine the viral infectivity as previously described [43]. Briefly, MAGI-CCR5 cells were cultured in 6-well plates one day before infection so that cells can reach approximately 40–50% confluency on the day of infection. The medium of each well was removed before the viral supernatant was added to infect cells in a total volume of 300 μl of complete DMEM with 20 μg/ml of DEAE-dextran. Cells in virus-containing medium were incubated at 37°C in a 5% CO_2 _incubator. After 2 hours incubation, 1.5 ml complete DMEM medium was added to each well. The cells were further incubated under the same condition for 48 hrs after incubation. The media were removed and 2 ml fixing solution (1% formaldehyde, 0.2% glutaraldehyde in PBS) was added to each well. Cells were washed twice with PBS and then fixed for 5 min. with 600 μl of staining solution (20 μl of 0.2 M potassium ferrocyanide, 20 μl of 0.2 M potassium ferricyanide, 2 μl of 1 M MgCl_2_, 10 μl of 40 mg/ml X-gal in PBS). Cells were then incubated for 2 hrs at 37°C in a non-CO_2 _incubator. Staining was stopped by removing the staining solution and washed twice with PBS. Blue dots were counted as infected cells as described previously [43] under the microscope. The viral infectivity was determined by comparing the total number of infected cells with uninfected cells in each well. Each experiment was performed in triplicate and results of these experiments were repeated at least three times. Statistic student *t*-test was used to determine potential significant difference among different treatment groups.

## List of abbreviations

HIV-1-human immunodeficiency virus type 1; Vif-viral infectivity factor; APOBEC3G- apolipoprotein B mRNA-editing enzyme catalytic polypeptide-like 3G; UBA2-ubiquitin-associated domain 2; UBL-ubiquitin-like domain; *p.i*.-post-infection; *p.t*.-post-transfection.

## Competing interests

The authors declare that they have no competing interests.

## Authors' contributions

LL and DL carried out all of the experiments. JYL provided supervision of the study and co-mentored LL's Ph.D. dissertation. RYZ supervised and directed the designed studies and co-mentored LL's Ph.D. dissertation. All authors read and approved the final manuscript.
